# Occurrence and spread of antibiotic-resistant bacteria on animal farms and in their vicinity in Poland and Ukraine—review

**DOI:** 10.1007/s11356-021-17773-z

**Published:** 2021-12-06

**Authors:** Karolina Jeżak, Anna Kozajda

**Affiliations:** Biological Safety Unit, Nofer Institute of Occupational Medicine, 8 Teresy Str, 91-348 Łódź, Poland

**Keywords:** Zoonotic bacteria, Antibiotic resistance microorganisms, ARG, Livestock, Farm’s vicinity, Livestock environment, Environmental exposure

## Abstract

Intensive animal farming emits to the environment very high concentrations of bioaerosol, mainly composed of microorganisms, including antibiotics resistant strains, and their derivatives. Poland is a significant producer of poultry and swine in Europe; Ukraine is located in the immediate vicinity of Poland and the EU. Thus, the review focuses on the presence of potentially pathogenic and antimicrobial-resistant zoonotic bacteria and antimicrobial genes in the environment of farms and food of animal origin in Poland and Ukraine. Existing data confirms presence of these bacteria in the food animal origin chain environment in both countries. However, it is difficult to compare the scale of multidrug-resistant bacteria (e.g. MRSA, ESBL) dissemination in Poland and Ukraine with other EU countries due to lack of more extensive studies and large-scale monitoring in these two countries. A series of studies concerning resistance of pathogenic bacteria isolated from livestock environment have been published in Poland but usually on single farms with a very limited number of samples, and without a genotypic drug resistance marking. From Ukraine are available only few reports, but also disturbing. The risk of antibiotic-resistant bacteria transmission does not only concern animal farming, but also other facilities of animal origin food supply chains, especially slaughterhouses.

## Introduction

The total number of bacterial species in all habitats in the world may exceed one million (Woolhouse et al. 2015). However, only about 20 species are known as typically human pathogens), and a slightly larger number (several hundred species) are opportunistic pathogens. Hundreds of other species are part of the normal human microflora, most of which are commensal bacteria that do not show any pathogenic properties, producing substances necessary for the proper functioning of the immune system and thus guaranteeing protection of the human body against the harmful pathogenic bacteria (Woolhouse et al. 2015, Tokarz-Deptuła et al. 2016). Antibiotic resistance of pathogenic bacteria causing bacteriemia increases morbidity and mortality, therefore is the challenge around the world. A particularly significant problem is the emergence of multi-drug resistance (Frieri et al. 2017, Akova 2016, Argudín et al. 2017, Woolhouse et al. 2015). The scale of antibiotic-resistant bacteria presence in the livestock environment now observed around the world is a consequence of widespread use of antibiotics et least decade earlier. Starting from the 1950s, antibiotics were used in agriculture mainly to prevent diseases, but also to promote growth, and increase animal production rates (as antibiotic growth promoters, AGPs). Soon, it became a common practice for veterinary doctors to prescribe antibiotics solely for that purpose, without indications for the treatment of bacterial diseases. Very often, the same antibiotics that were used in agriculture and in veterinary were also used in the treatment of people (Woolhouse et al. 2015; Zabłotni and Jaworski 2014). For treating purposes, they should only be administered to animals with a confirmed infection. However, it is a common practice to administer antibiotics to the entire herd or flock (for metaphylactic purposes). Wanot and Domagała (2019) report that the use of prophylactic doses of antibiotics in poultry, cattle and pig breeding is much greater than for therapeutic purposes. In 2014, 581 tonnes of antibiotics were sold for use in veterinary medicine in Poland. Used in large quantities, antibiotics, which are present also in animal feed and water, may accumulate in the tissues and meat of the farmed animals. Since 2014, the General Veterinary Inspectorate in Poland has been monitoring the drug resistance of the zoonotic bacteria: *Salmonella, Escherichia coli* and *Campylobacter*. The results clearly indicate a rise in the drug resistance of microorganisms (Wanot and Domagała 2019).

The consequence of intensive animal production is a high level of pollution emitted to the environment, the nearest vicinity of farms, but also air, soil, surface water, groundwater, and rainwater (Woolhouse et al. 2015; Gordon, 2018; Augustyńska-Prejsnar et al. 2018; Wanot and Domagała 2019). The use of liquid manure as fertiliser poses the risk of polluting the environment with pathogens, antibiotics, metabolites of antibiotics and antibiotic-resistant pathogens (Woolhouse et al. 2015; Gordon 2018; Argudín et al. 2017; Skowron et al. 2015; Wanot and Domagała 2019).

Among the pathogenic zoonotic bacteria on animal farms environment are: *Staphylococcus* spp.; *Salmonella* spp.; *Campylobacter* spp.; E*. coli*; *Listeria* spp.; *Enterococcus* spp. These pathogens are emitted into the air (organic dust) and through surface waters (slurry, faeces of other animals, including poultry) to the environment, where first as viable cell can be a cause of infections in humans and second constitute the source of antibiotic resistance genes (ARG) (Argudín et al. 2017). The excessive use of antibiotics in veterinary, until the limiting regulations were forced, resulted mainly from the use of them as AGPs in livestock (Bengtsson and Wierup 2006; Aarestrup et al. 2001). Now, in the EU and almost all over the world, these issues are regulated but studies conducted on animal farms undoubtedly indicate that antimicrobials are abused (Giedrojć-Brzana et al. 2017; Zalewska et al. 2017; Wanot and Domagała 2019). Currently, it is difficult to assess the practice in animal farms in Poland, because there is a certain inconsistency between such antimicrobial use reports and the strict supervision system over the use of these drugs in animals which is in force in Poland (Chef Veterinary Officer 2019). In Ukraine, an assessment of use of antibiotics in animal farming in practise is even more difficult due to the small availability of information.

Large amounts of antibiotics used in medicine and veterinary have resulted in the selection of pathogenic multi-antibioticresistant bacteria. The presence of a single resistance mechanism does not ensure the survival of bacteria, therefore it is common for bacteria to develop several various resistance mechanisms to different groups of antibiotics simultaneously (Wasążnik et al., Nikaido 2009). In literature are described many mechanisms of antibiotic resistance, but all of them are based on several strategies. The most common are: production of antibiotics-inactivating enzymes, blockade of target sites for the antimicrobials, change in cell membrane permeability and active antimicrobial efflux from cell. Bacterial multi-antibioticresistance can be generated by one of two mechanisms. The first consists in accumulation of many genes coding the resistance to a single drug in an individual bacterial cell. This type of multi-antimicrobial resistance usually occurs in R plasmids.The second multi-antibioticresistance mechanism consists in increased expression of genes encoding transport proteins (efflux pumps), each of which may actively pump out of the cell to the external environment one or more type of drugs / substances (antibiotics, dyes, detergents, toxins and active substances of disinfectants and antiseptics). The genes responsible for efflux pumps are usually coded on the chromosome (e.g. the genes encoding the AcrAB-TolC system), but they can also be present on plasmids and other mobile genetic elements (e.g. the qac and tet genes) (Wiercińska et al. 2015; Nikaido 2009; Nikaido and Pagès 2012; Kim et al. 2021).

In the European Union, from the January 2006, pursuant to Regulation No. 1831/2003 of the European Parliament and of the Council of On August 22, 2003, the marketing and use of antibiotics as feed additives is prohibited. In Poland veterinarians providing veterinary services are responsible for keeping trading medicines records and veterinarian documentation. Law in force requires documentation of the trading of all prescription veterinary medicinal products intended for use in both farm and domestic animals (Giedrojć-Brzana et al. 2017).

Currently, the use of antibiotics to promote growth in livestock and poultry is prohibited in the EU as well as in Ukraine. Antibiotics can be administered to animals only in justified cases, and only when prescribed by and under the supervision of a veterinarian. However, various authors suggest that the introduction in the EU in 2006 of the growth promoters prohibition only slightly reduced the use of antimicrobials, the subtherapeutic use was replaced with metaphylaxis and prophylaxis (Argudín et al. 2017; Woolhouse et al. 2015; Wanot and Domagała 2019). The costs of producing medicated feed on a farm are very high, and meeting the veterinary requirements is usually difficult for small and medium agricultural holdings. It is possible, then, that farmers add the drugs provided by veterinarians to the feed without following the requirements. In practice, pharmaceutical and veterinary inspections often lack adequate tools to prevent the illegal trade in veterinary drugs (Giedrojć-Brzana et al., 2017; Wanot and Domagała 2019). This thesis is confirmed by the results of the audit carried out by the Supreme Audit Office in Poland in the Lubuskie Voivodeship indicating that antibiotics are commonly used in animal farms. This conclusion was based on measurements of the antimicrobial substances presence in water and feed, which indicated that antibiotics were used in 70% of monitored farms. Relevant is that in turkeys and broilers farms this percentage was even higher, exceeded 80%. Results of the monitoring indicates that scale and scope of the antibiotics use raise legitimate concerns about the effects on public health now and in the future (Supreme Audit Office 2018).

In this review, we focus on the results of studies on the presence of potentially pathogenic and antimicrobial-resistant zoonotic bacteria and antimicrobial genes in the environment of farms and food of animal origin carried out in Poland and Ukraine.

## Methods

The literature was structurally reviewed in the PubMed database using individual crossings of the following key words: ‘livestock’, ‘animal farms’, ‘animal breeding’, ‘environment’, ‘antimicrobial resistance’, ‘AMR’; ‘horizontal gene transfer’, ‘resistance genes’, ‘AMR-gene’, ‘farm vicinity’, ‘Poland’, ‘Ukraine’ in years 2000–2020 (December). There was performed a two-step search strategy, first, PubMed was searched by above key words and next the reference lists of all identified articles were searched for additional eligible studies. Inclusion criteria was strictly defined: published scientific and grey literature, reports published by EU and national authorities, national governmental institution or national inspections. The exclusion criteria were studies conducted outside Poland and Ukraine and studies carried out on food of animal origin purchased in stores due to the possibility of cross-contamination. The reliability of the articles and reports was assessed by verifying the source (publishers and affiliation of authors).

## Results

### Research on farm animals and their surroundings

Review of the literature on presence of antimicrobial-resistant pathogen bacteria and ARG in the environment of animal farm and their vicinity in Poland and Ukraine is presented in Table 1.

**Table 1:** Presence of ARB and ARG in the environment of animal farm in Poland and Ukraine

Type and number of farms	Samples source, type and number	Identification method	Identified species / strains	Methods for determining antibiotic resistance	Resistance to antibiotics	ARGs	Reference (country)
5 turkey farms; 2 chicken farms	45 swabs fresh poultry faeces,	molecular	*Campylobacter jejuni, Campylobacter coli* In total were identified 45 strains of *Campylobacter* genus, including 31 from the turkey faeces and 14 from the broiler faeces. Among them 41 strains (91.1%) were identified as *C. jejuni*, and 4 strains (8.9%) as *C. coli*. The turkey samples were the source of 30 *C. jejuni* strains and 1 *C. coli* strain, while the number of *C. jejuni* and *C. coli* isolates from the broiler faeces was 11 and 3, respectively	minimum inhibitory concentration (MIC); molecular methods	Strains susceptible to azithromycin, erythromycin, gentamicin, florfenicol, telithromycin, clindamycin; all strains resistant to ciprofloxacin; 92.9% strains from broiler resistant to nalidixic acid and 78.6% strains resistant to tetracycline; 83.9% strains from turkey resistant to nalidixic acid and 58.1% % strains resistant to tetracycline	all strains resistant to ciprofloxacin had a mutation in the *gyr*A gene at Thr-86 position; presence of the *tet*O gene in 71% of isolates from the turkey and in100% of isolates from the broiler; all strains resistant to tetracyclines contained the *tet*O gene; 5 strains from turkey faeces and 3 strains from broiler faeces susceptible to tetracycline contained the *tet*O gene	Woźniak-Biel et al. 2018 (PL)
3 poultry farms	various waste materials collected on poultry farms: 4 feather samples (duck, turkey, chicken), sludge (2 samples) centrifuge sediment (2 samoles)	selective medium	100 strains of *Enterococcus genus* identified as *Enterococcus faecalis* (21%)*, Enterococcus faecium* (79%)	Chromogenic differentiating medium ChromID VRE; disc diffusion method	More than 50% of the strains demonstrated resistance to 19 out of 23 antibiotics. The highest resistance in the case of streptogramins, carbapenems, fluoroquinolones, aminoglycosides, penicillins; the lowest in the case of nitrofurantoin and chloramphenicol; *E. faecalis* strains were more resistant than the *E. faecium;* presence of multidrug resistance of isolates	-	Cybulska and Krzyśko-Łupicka 2020 (PL)
	environmental samples from chicken broilers, turkey broilers and laying hens; 51 isolates from 2 veterinary laboratories originated from livestock environment;	n/a	*Salmonella enterica* ser. Enteritidis	minimum inhibitory concentration (MIC); molecular methods	all isolates susceptible to gentamicin, tazobactam, cefotaxime, meropenem, azithromycin, tigecycline and trimethoprim, ampicillin, 84.3% isolates resistant to colistin, 56.9% to sulfamethoxazole; 41.2% to nalidixic acid, 21.6% to tetracycline, 3.9% to chloramphenicol, 2.0% to ciprofloxacin	14 isolates showing multidrug resistance; resistance genes: *flo*R (51.0%), *bla*TEM (35.3%), *bla*PSE (5.9%), *tet*A (37.3%), *tet*B (17.6%), *tet*C (2.0%), *tet*G (3.9%), *cat*1 (11.8%)	Ćwiek et al. 2020 (PL)
	feed samples provided as part of official controls; 452 animal feed samples (complete feedingstuff, premixtures, concentrates, feed additives, and water, 126 flush material samples (mixtures or feed materials used to flush the production line after producing a batch of medicated feed);	n/a	n/a	agar well diffusion method (microbiological diffusion screening method based on the growth inhibition of selected test strains)	antibacterial substances confirmed in 185 samples (57 samples of flush material, 127 samples of feed, 1 sample of water)	-	Przeniosło-Siwczyńska and Kwiatek 2013b (PL)
poultry and pig farms	samples from, animal feed, and sources, such as food and sewage sludge	n/a	560 *Salmonella* isolates; 42 different serotypes of *Salmonella*	ATBVET diagnostic kits	12.9% of isolates susceptible to all 28 antimicrobial substances; resistance in 488 (87.1%) of isolates; 37.3% strains resistant to few antimicrobial substances; 49.8% strains were multiresistant; bacteria originating from pigs showed more resistance than from poultry and feed samples; Multidrug resistance observed in the majority of S. Typhimurium, S. Hadar and S. Gallinarum strains	-	Wasyl and Hoszowski 2004 (PL)
	samples from turkeys, turkey or unspecified poultry meat, food production hygiene checks, feed, municipal sewage sludge	n/a	27 *Salmonella enterica* serotype Kentucky isolates	minimal inhibitory concentration (MIC); molecular methods	fluoroquinolone resistance in 25 isolates; one isolate was extended spectrum β-lactamase- (ESBL) positive; 1 isolate resistant to cefotaxime and ceftazidime, remaining isolates susceptible to this antibiotics	multiple mutations within chromosomal genes *gyr*A and *par*C responsible for high-level ciprofloxacin resistance; presence of *bla*_CTX-M-25_ gene and an integron with another β-lactamase encoding gene—*bla*_OXA-21_	Wasyl et al. 2015 (PL)
	9670 isolates from animals, food, and feed	n/a	2680 *Salmonella* isolates; 136 *Salmonella* serovars	minimal inhibitory concentration (MIC); molecular method	6.4% of 2680 isolates resistant to ciprofloxacin	PMQR (plasmid mediated quinolone resistance) mechanisms observed in 92 (3.4%) isolates; PMQR mechanisms in 11 Salmonella serovars identified as *qnr*S1 or *qnr*S3 and *qnr*B10 or *qnr*B19; *qnr*S1/S3 and *qnr*S2 identified in 2 isolates with chromosomal mutations; chromosomal substitutions in QRDR (quinolone resistance determining region) found in *gyr*A and *par*C	Wasyl et al. 2014 (PL)
	milk samples from cows with mastitis	n/a	123 strains of *Staphylococcus aureus* isolated	disk diffusion method; minimal inhibitory concentration (MIC); molecular method	86 isolates susceptible to all 20 tested antibiotics (70%); 22 isolates resistant to amoxicillin (17.9%), 28 to ampicillin (22.8%), 29 to penicillin (23.6%), 13 to streptomycin (10.6%); all strains resistant to at least one antibiotic (n = 37) and two strains susceptible to all chemotherapeutics susceptible to lysostaphin, 21 of them resistant to nisin; 30 isolates resistant to two or more (up to 6) of the tested antibiotics	25 of the penicillin-resistant strains were found to carry the *bla*Z gene coding for β-lactamases; 2 strains *mec*A positive	Szweda et al. 2014 (PL)
dairy farm	717 milk samples from 583 cows with clinical and subclinical mastitis;	n/a	12 strains of MRSA	disc diffusion method;	5 isolates of *S. aureus* identified as MRSA; all isolates (n = 5) susceptible to amikacin, chloramphenicol, cotrimoxazole, fusidic acid, gentamicin, and mupirocin, and resistant to ciprofloxacin, clindamycin, norfloxacin, and tetracycline	-	Krukowski et al. 2020 (PL)
4 farms	81 nasal swabs from 42 riding horses	CHROMagar MRSA medium, molecular method	In total were isolated 87 staphylococci, including 11 species of coagulase-negative *Staphylococci* (CoNS) 82.8% CoNS were identified using the PCR–RFLP method. Majority of strains were identified to species (82.8%), from which the most numerous were: *‒ S. equorum* (14.9%), *‒ S. warneri* (14.9%), *‒ S. sciuri* (12.6%), *‒ S. vitulinus* (12.6%), *‒ S. xylosus* (11.5%)	molecular method	17.2% of the CoNS isolates resistant to 1 or 2 out of 18 antimicrobial agents; 17 isolates of methicillin-resistant coagulase-negative Staphylococci (MRCoNS)	*mec*A gene detected in all 5 cefoxitin-resistant isolates and in 12 cefoxitin-susceptible isolates	Karakulska et al. 2012 (PL)
6 turkey farms	62 samples of fresh faeces or cloacae swabs from 22 healthy turkeys	Maldi-Tof and molecular method	*Lactobacillus* strains were detected in 53 out of 62 samples taken. In total identified 62 isolates of *Lactobacillus* belonging to 8 species, most frequently: *- L. salivarius* 22 strains (35%); *- L. crispatus* 11 (21%); *- L. ingluviei* 9 (14.5%); *- L. johnsonii / L. gasseri* 5 (10%)	broth microdilution procedure using the LAB susceptibility test medium (LSM); molecular method	68% isolates resistant to tetracycline, 60% to enrofloxacin, 47% to ampicillin, 45% to erythromycin, 31% to streptomycyn, 29% to chloramphenicol, 10% to gentamicin; 6% of all isolates resistant to streptomycin and gentamicin simultaneously; in 43.5%a cross-resistance between erythromycin and lincomycin; multidrug resistance confirmed in 64.5% isolates	presence of antibiotic resistance genes was related to phenotypic resistance except for 5 sensitive isolates which contained *tet*M, *tet*L, *erm*C, *erm*B or *cat* genes; most frequently identified genes were *erm*B (45%), *tet*L (40%), *tet*W (37%) and *tet*M (29%)	Dec et al. 2018 (PL)
14 geese farms	fresh faeces from 50 birds	Maldi-Tof and molecular method	93 isolates of *Lactobacillus* belonging to 10 species, the most abundant were following: *- L. salivarius* (n = 35) *- L. johnsonii* (n = 17) *- L. ingluviei* (n = 10) *- L. agilis* (n = 8)	minimal inhibitory concentration (MIC)	100% isolates sensitive to ampicillin and amoxicillin; 52 (56%) isolates resistant to flumequine, 25 (26.9%) to neomycyn, 22 (23.6%) to tetracycline, 21 (22.6%) to enrofloxacin, 17 (18.3%) to doxycycline, 14 (15%) to lincomycin, 9 (9.7%) to tylosin; 72 (77.4%) strains resistant to at least one substance;11 (11.8%) strains were multidrug resistance; cross-resistance to tetracycline and doxycycline in 17 (18.3%) isolates, to enrofloxacin and flumequine in 16 (17.2%); simultaneous resistance to tylosin and lincomycin in 7 (7.5%) strains Furthermore, different species of *Lactobacillus*, isolated from the same sample, exhibit acquired resistance to the same antibiotic	-	Dec et al. 2015 (PL)
12 poultry farms	293 hen manure samples	-	-	liquid chromatography with a Waters double mass spectrometric detector	presence of antibiotics in 112 of samples (38.2%); in 54.8% of positive samples detected antibiotic from the tetracycline group (doxycycline, oxytetracycline, tetracycline, and chlortetracycline); in 39.8% of positive samples detected fluoroquinolones (enrofloxacin and norfloxacin); penicillines detected in 3.57% of positive samples (amoxicillin and florfenicol)	-	Shevchenko et al. 2019 (UA)
5 dairy farms i	165 samples of varius origins: 32 samples of raw milk, 38 swabs of udder skin, 38 samples of milk from cows with subclinical mastitis and 57 environmental samples (10 swabs of milking machines, 13 swabs of milk tanks, 10 samples of animal feed, 24 swabs from floors of farm buildings)	n/a	62 isolates *of Staphylococcus aureus*	disk diffusion metod; molecular method	Isolates resistant to penicillin (n = 50, 80.6%), oxacillin (n = 33, 53.2%), lincomycin (n = 16, 25.8%), tetracycline (n = 18, 29.0%), ciprofloxacin 9.9% (6/62) and streptomycin 4.8% (3/62); 20 isolates (32.3%) resistant to penicillin and oxacillin were also resistant to vancomycin; all isolates susceptible to gentamicin, enrofloxacin and erythromycin; 31 isolates resistant to 3 antibiotics, 13 isolates to 4 antibiotics, 15 isolates to 5 antibiotics	15 isolates of *S. aureus* had *mec*A gene	Berhilevych et al. 2017 (UA)

*PL* Poland; *UA* Ukraine; *n/a* not available

In order to verify whether the regulations on the use of veterinary medicinal products are being followed by individual farmers running agricultural holdings, an anonymous survey was conducted (Giedrojć-Brzana et al. 2017) among 613 farmers from the Wielkopolska and Silesia regions. The analysis demonstrated that a large percentage of drugs used on pig farms was added to feed. It was also noted that the larger the holding was, the more infrequent was the use of drugs of unknown origin (Giedrojć-Brzana et al. 2017). The costs of producing medicated feed for a specific herd at a specific time, with ingredients prescribed by a veterinarian, are very high. For small and medium agricultural holdings, meeting all veterinary requirements is generally difficult. It is possible, then, that farmers add the drugs provided by veterinarians to the feed without following the requirements (Giedrojć-Brzana et al. 2017).

*Salmonella enterica* serotype Enteritidis (*S. enterica* ser. Enteritidis) is one of the salmonella serotypes most frequently detected in humans. These bacteria have a capacity to form biofilms, and the risk of transmission from animals and foods of animal origin to humans is substantial.

Mazurek et al. (2015) studied the prevalence to resistance to trimethoprim and sulfamethoxazole occurred in *E. coli* strains isolated from a pig farm located in western Poland. The *E. coli* strains were isolated from faeces samples originating from three groups of piglets at different stages of metaphylactic treatment and from two groups of sows that had been subjected to the same metaphylactic treatment in the past (6-week-old piglets after the first week of treatment, 7-week-old piglets after the second week of treatment, 8-week-old piglets after the third week of treatment, sows (1) 10 weeks after the end of treatment, and sows (2) 18 weeks after the end of treatment). The treatment was prescribed for colibacillosis. The antimicrobial drugs were administered in the form of medicated feed containing trimethoprim/sulfamethoxazole. The resistance of the *E. coli* isolates to these antibiotics was assessed using the EUCAST standards. The study also examined the presence of resistance genes (*dfr*A1, *dfr*A5, *dfr*A7, *dfr*A12, *dfr*A17*, sul*1, *sul*2, *sul*3) and *dfr*A gene cassettes using the PCR method. In total, 352 bacteria strains were examined. The majority of the isolates were resistant to the antimicrobial drugs. Between 97 and 100% of strains isolated from piglets, 86% of strains isolated from sows (1), and 69% of strains isolated from sows (2) showed resistance to trimethoprim. All strains isolated from piglets and sows (1), and 92% of strains isolated from sows (2) were resistant to sulfamethoxazole. In the case of piglets, the most frequently detected trimethoprim resistance gene was the *dfr*A1 gene. The co-occurence of different *dfr*A genes was confirmed in 71 isolates from all groups of pigs. In strains isolated from sows, *dfr*A1 and *dfr*A12 genes occurred with a similar frequency. *dfr*A7 and *dfr*A5 genes were detected in strains from all groups, but with a much lower frequency. None of the isolates under study contained the *dfr*A17 gene. The dominant sulfamethoxazole resistance gene in the strains isolated from piglets was the *sul*1 gene, and in the case of sows, it was the *sul*3 gene. The *sul*2 gene occurred less frequently in strains from all groups. The study also confirmed the co-occurence of multiple resistance genes in individual strains. Moreover, the authors stated that a significant number of *dfr*A genes present in the majority of the *E. coli* isolates, including the most frequently identified *dfr*A1 gene, had not undergone transcription (therefore, were not expressed) (Mazurek et al. 2015).

In the study conducted by Krukowski et al. (2020), the first epidemic of Livestock-associated meticillin-resistant *Staphylococcus aureus* (LA-MRSA) in dairy cattle, as well as the first probable case of MRSA transmission between humans and cows in Poland were presented. The authors conducted the study on a farm specializing in milk production, located in eastern Poland. Samples were collected in order to check the cows for mastitis. During 1-year study period, 717 samples from 583 cows were collected and microbiologically tested. A total of 5 isolates of MRSA were cultured from the samples collected from cows with asymptomatic mastitis. In the same time, 24 oral and nasal swabs were collected from 6 people: the dairyman, veterinarian and 4 of their family members. A total of 8 swabs (taken from the dairyman, the veterinarian and 2 members of the veterinarian’s family) tested positive for MRSA. DNA sequencing was performed on all of the MRSA isolates. Eleven of them (5 bovine and 6 human) were revealed to have identical antibiotic sensitivity profile and were of clonal lineage ST398, spa *t*034 type. The authors presented evidence for transmission of MRSA between the cattle and the farm workers and between people in a family environment. The risk of intra- and interspecies transmission of LA-MRSA was confirmed (Krukowski et al. 2020).

The problem of processing, use and disposal of poultry manure contaminated with antibiotics remains unsolved not only in Ukraine but worldwide, and the treatment and prevention of highly contagious infectious diseases in poultry requires the use of antibacterial medication.

A major problem in milk production is staphylococci, which in many ways enter raw milk and dairy products during their production. Bacteria of the species *S. aureus* can live on the surface of the skin of cow udders or in the teat canals. In case of infection with these bacteria, the mammary glands are the main source of contamination of raw cow milk, dairy equipment and the entire dairy farm environment. These bacteria are important for human health, especially, as they are present in both raw milk and the dairy farm environment, they may be potential carriers of antibiotic resistance genes (Berhilevych et al. 2017).

### Studies of airborne and settled dust inside breeding facilities in Poland and Ukraine

Dust in livestock premises has high concentration of microorganisms, including those that are antibiotic-resistant. The main factors affecting the environmental conditions inside and outside of animal buildings include concentration and type of animal production, breeding system, organisation of the production process, indoor microclimate and the quantity and quality of animal faeces. People working in intensive animal farming are exposed to high concentrations of organic dust and suspended biological agents. The average concentrations of organic dust for employees working directly with pigs may exceed 12 mg/m^3^ and 0.3 mg/m^3^ of air for inhalable and respirable fractions respectively (Buczyńska and Szadkowska-Stańczyk 2010; Szadkowska-Stańczyk et al. 2010). Szadkowska-Stańczyk et al. (2010) conducted a study at swine farms located in Poland. During the study involved 90 individual measurements of airborne dust collected with pumps attached to clothes of swine farm workers during their work in livestock buildings. The analysis of dust concentrations was done with division into inhalable and respirable fractions. The analysis of samples showed that the concentration of inhalable dust raged from 0.16 to 37.2 mg/m^3^ of air and of respirable dust from 0 to 4.28 mg/m^3^. The determined concentration values were compared with Polish occupational exposure limit (OEL) values for this type of dust: 4 mg/m^3^ for inhalable dust and 2 mg/m^3^ for respirable dust. The analysis showed that the OEL values were exceeded in 47% and 7% of cases, respectively for the concentrations of inhalable and respirable fractions. The concentrations of bacterial microflora in piggery air exceeded the levels recommended in Poland as safe for workers’ health. Total concentrations of microorganisms in the air of piggeries covered by the study were high and ranged from 4.38 × 10^4^ to 1.07 × 10^6^ cfu/m^3^ (colony-forming units in m^3^ of air). Bacteria accounted for over 96% of detected microorganisms, with the mean concentration of 4.79 × 10^5^ cfu/m^3^ of air (Szadkowska-Stańczyk et al. 2010).

Sowiak et al. (2012), in a study conducted in buildings of swine farms located in Poland, found concentrations of airborne dust ranging from 0.48 to 9.82 mg/m^3^ and from 0.08 to 4.69 mg/m^3^ respectively for the inhalable and respirable fraction. The authors of the study also found that the factors significantly affecting the total concentration of bacteria, as well as their levels in the inhalable fraction of dust are herd size, breeding system, feeding method, type of ventilation and airflow velocity. Higher concentrations of bacteria were observed in cases of small herds, litter bed system, manual feed distribution, natural ventilation and low airflow velocity (Sowiak et al. 2012).

The air in poultry houses at poultry farms is usually heavily contaminated by large quantities of dust particles of biological and non-biological origin. Bioaerosol in poultry houses contains particles released chiefly from settled dust, which originates from feed, manure, litter, feather fragments and animal skin, as well as microorganisms, their bioproducts and fragments (Ławniczek-Wałczyk et al. 2013).

Ławniczek-Wałczyk et al. (2013) conducted a study on poultry farms to determine the concentrations of PM_10_ and the level of microorganisms in it. The analysis showed that the PM_10_ concentrations ranged from 73 to 4095 μg/m^3^ in winter and from 28 to 4511 μg/m^3^ in summer. The range of total concentrations of microorganisms in air of poultry houses was 2.1 × 10^5^–2.1 × 10^11^ cfu/m^3^ in winter and 1.1 × 10^4^–3.6 × 10^12^ cfu/m^3^ in summer (Ławniczek-Wałczyk et al. 2013).

Another study, conducted on 13 poultry farms located in the Kujawsko-pomorskie and Łódzkie voivodeships, determined concentrations of airborne and settled dust. The settled dust was also analysed microbiologically. PM_10_ was found to be the predominant fraction of dust collected on poultry farms (average concentration: 0.875 mg/m^3^, maximum: 2.128 mg/m^3^). Dust particles with lower diameters PM_1_, PM_2.5_, PM_4_ were found at levels ranging from 0.480 to 0.541 mg/m^3^. Concentration of bacteria in settled dust ranged from 1.5 × 10^7^ to 1.7 × 10^7^ cfu/g dust. Qualitative analysis of the dust found, i.e., bacteria belonging to the genera *Enterococcus* and *Salmonella*, as well as to the *Escherichia coli* species (Skóra et al. 2016).

Bródka et al. (2012), in a study conducted on a poultry farm, found concentrations of airborne bacteria ranging from 4.74 × 10^4^ to 1.89 × 10^8^ cfu/m^3^. Most of the bacteria were gram-positive microorganisms, with more than 40% of them classified as bacteria of *Enterococcus* genus.

The aim of the study conducted by an international team of Tsapko et al. (2011) was to compare bioaerosol concentrations at cow and pig farms and at animal feed facilities in Ukraine and Poland. In the studied facilities, concentrations of dust and microorganisms in the air inside working zones were determined. The studies in Ukraine were conducted between 1980 and 2009 in 4 intensive cattle breeding, 4 dairies, 2 piggeries and 5 animal feed facilities. It was found that dust concentrations ranged between 6 and 200 mg/m^3^ of air in animal houses and between 35 and 306 mg/m^3^ of air in feed facilities. The highest dust concentrations were found at loading of fodder, feeding of animals, and removing manure. The concentrations of airborne microorganisms in animal buildings ranged from 5.5 × 10^4^ to 1.90 × 10^7^ cfu/m^3^ of air. In animal feed facilities they ranged from 2.70 × 10^4^ to 2.60 × 10^9^ cfu/m^3^. In Poland, studies were conducted at 4 cow farms, 10 piggeries and in 1 large animal feed facility. Dust concentrations at cow farms ranged between 0.25–0.80 mg/m^3^, in piggeries between 3.03–14.05 mg/m^3^, and in the animal feed factory between 3.8–405.0 mg/m^3^ of air. The concentrations of microorganisms in airborne dust ranged between 4.70 × 10^4^–2.90 × 10^5^ cfu/m^3^ at cow farms, between 6.0 × 10^5^–1.50 × 10^6^ cfu/m^3^ at pig farms, and between 1.70 × 10^3^–2.0 × 10^6^ cfu/m^3^ of air at animal feed facilities (Tsapko et al. 2011).

### Antibiotic-resistant bacteria in carcasses, meat, internal and external environment of slaughterhouses

Bacteria of the genus *Campylobacter* and *Salmonella* can be transmitted to humans through food (one of the significant sources is poultry) and cause gastritis and enteritis (Wysok et al. 2015; Mąka et al. 2015). Bacteria of the *Enterobacteriaceae* family are a natural part of the intestinal microflora of humans and animals. They are mostly symptomless or opportunistic species, however, there is also a group of pathogens which are responsible for a significant number of food infections and various gastrointestinal complaints. A characteristic feature of Gram-negative bacteria of the *Enterobacteriaceae* family is their high capability of acquiring antibiotic-resistance genes, which is passed on to related genera of bacteria (Szewczyk et al. 2019). Methicillin-resistant *S. aureus* (MRSA) strains are a significant problem in epidemiology around the world, and their origin is largely in close relationship with intensive animal farming (Krupa et al. 2015).

Data regards presence of antibiotic-resistant bacteria in carcasses, meat, internal and external environment of slaughterhouses in Poland and Ukraine are presented in Table 2.

**Table 2:** Presence of antibiotic-resistant bacteria in carcasses, meat, internal and external environment of slaughterhouses in Poland and Ukraine

**Bacteria**	**Source (samples number)**	**Identification method**	**Antimicrobials susceptibility analyse method**	**Main results**	**Author**
*Campylobacter jejuni*; *Campylobacter coli*	Chicken carcasses from stores (n = 130); stool samples of child patients with diarrhea obtained from three large paediatric hospitals (n = 946)	molecular	Etest (Epsilometer test)	In total, there were 149 isolates of *C. jejuni* (60 carcasses; 89 stool) and 54 isolates of *C. coli* (40 carcesses; 14 stool). All *Campylobacter* strains isolated from carcasses and all (except one) isolated from humans were susceptible to macrolides. Almost all of them were also susceptible to aminoglycosides. Resistance to gentamicin was found in three *C. jejuni* strains isolated from stool from child patients and one strain isolated from meat. Most commonly, in the case of human isolates and chicken carcass isolates alike, drug resistance was observed to ciprofloxacin (over 40% isolates), with no significant fluctuations observed over the course of the study. Between 2003 and 2005, resistance to ampicillin increased from 8% to 35.5% in human-derived isolates and from 5.8% to 30.4% in isolates coming from chicken carcasses. General resistance to tetracycline in strains of *C. jejuni* and *C. coli* isolated from chicken carcasses was 8.3% and 10%, respectively. For isolates from stool samples, the resistance was 15.7% and 28.5% respectively. According to statistical analysis taking into account sampling dates, resistance to tetracycline in cultures isolated from chicken carcasses increased from 0% in 2003 to 17.3% in 2005. Further, all quinolones-resistant strains exhibited cross-resistance to nalidixic acid and ciprofloxacin. Resistance to two drugs was more frequent in cultures from stool (27 isolates) than in those from chicken meat (7 isolates). Two *C. jejuni* isolates from stool were simultaneously resistant to four antimicrobial agents. All tetracycline-resistant cultures had the tat (O) gene and all quinolones-resistant cultures according to the E-test had the Thr-86-Ile mutation in the *gyr*A gene. The slight differences in resistance between human- and poultry-derived isolates indicate that although chicken meat is not the only source for *Campylobacter* infection, it can be involved in transmitting drug-resistant strains to humans	Rożynek et al. 2008 (PL)
*Campylobacter* spp.	bovine carcasses (n = 144); pork carcasses (n = 177); both type of carcasses form various slaughterhouses	molecular	-	In total of 70 isolates with *Campylobacter* were isolated. The prevalence of Campylobacter in bovine and pork carcasses samples was 17 (14.9%) and 53 (29.9%), respectively. Most beef carcasses were infected with *C. jejuni* (11 isolates; 64.7%) while pork carcasses were mostly positive for *C. coli* (41 isolates; 77.4%). All analysed strains were resistant to gentamicin and chloramphenicol. It was found that Campylobacter bacteria were most frequently resistant to quinolones (nalidixic acid and ciprofloxacin, 57.1% in total), streptomycin (52.9%, only C. coli strains) and tetracycline (51.4%). Resistance to erythromycin was confirmed only in four strains of C. coli from pork carcasses. Multidrug resistance (to at least two different classes) was confirmed in 43 (out of 70) isolates, mostly C. coli; a majority of them derived from pork (35 isolates). Multidrug-resistant strains exhibited resistance from 2 to 5 different antibiotics	Wieczorek and Osek 2013 (PL)
*Campylobacter* spp.	poultry (n = 71); pigs (n = 174); cattle (n = 277); Samples from animals were obtained in a slaughterhouse from small intestine contents and carcass swabs Human faeces samples (n = 1347) from patients with gastrointestinal symptoms collected in laboratory	molecular	disc diffusion method	*Campylobacter* spp. was isolated from poultry (100%; 91.5%) pigs (51.5%; 29.9%) and cattle (36.4%; 10.5%) from the small intestine contents and carcases swabs, respectively*.* In humans material *Campylobacter* spp. was found in only 3.4% samples In material isolated both from humans and slaughter animals confirmed only strains of *C. jejuni* and *C. coli* Resistance to eight antibiotics (erythromycin, gentamicin, ciprofloxacin, ampicillin, tetracycline, chloramphenicol, doxycycline and nalidixic acid) was evaluated. In total, 141 isolates of bacteria obtained from swine, 136 isolates from poultry, 130 isolates from cattle and 46 isolates from human were tested. All *Campylobacter* strains isolated from animals were susceptible only to chloramphenicol. High susceptibility to gentamicin (100% isolates from poultry and swine and 94.6% from cattle) and erythromycin (100% isolates from cattle, 94.3% from swine and 94.0% from poultry) was also confirmed In isolates from animals was found a higher level of resistance to ampicillin (range: from 20.8% in cattle to 42.3% in poultry). Resistance to quinolone and fluoroquinolone antibiotics turned out to be the highest. There was confirmed in isolates of poultry, swine and cattle resistance to: - ciprofloxacin: 64.5%, 61.7% and 53.9%; - nalidixic acid: 71.4%, 51.8% and 60.0%; - tetracyclin: 59.3%, 56.2% and 64.5%; - doxycycline: 55.6%, 70.0% and 65.3%; respectively Among the *Campylobacter* strains isolated from humans, the more than half (56.6%) was resistant to three or more antibiotics. Susceptibility to all of the tested antibiotics was confirmed only in 5.4% isolates. In this group of isolates was found resistance to: doxycycline (58.7%), tetracycline (56.5%), ciprofloxacin (58.7%), nalidixic acid (63.1%), erythromycin (8.7%) and gentamicin (10.9%) Among animal samples, susceptibility to all of the tested antibiotics was confirmed in 4.2%, 3.8% and 2.9% in swine cattle and poultry. There was noted also strains resistant to three or more of the tested antimicrobial agents	Wysok et al. 2015 (PL)
*Campylobacter* spp.	Chicken giblets and meat samples (n = 218)	n/a	n/a	*Campylobacter* bacteria was confirmed in 65.6% of samples (*C. coli* 75.5% and *C. jejuni* 24.5%). Isolates were tested on susceptibility to antibiotics: fluoroquinolones (ciprofloxacin), macrolides (erythromycin), tetracyclines (tetracycline) and aminoglycosides (gentamicin). The analysis confirmed a high rate of ciprofloxacin-resistant *Campylobacter* isolates (73.4%), erythromycin (64.3%) and gentamicin (6.3%). Among 143 resistant *Campylobacter* strains, 7.0% was resistant to at least three antibiotics	Maćkiw et al. 2012 (PL)
*Campylobacter jejuni*; *Campylobacter coli*	Skin and carcasses samples of 406 slaughtered cows from 3 slaughterhouses (n = 812 samples)	molecular		*C. jejuni* and *C. coli* *Campylobacter* strains were isolated from 25.6% and 2.7% of skin and carcass samples, respectively. Among the isolates from cattle skin 58.7% was identified as *C. jejuni* and 41.3% as *C. coli*, whereas among the strains isolated from cattle carcasses, 63.6% was identified as *C. jejuni* and 36.4% as *C. coli* Susceptibility analysis of *Campylobacter* isolates revealed that 49.6% were sensitive to all of the tested antibiotics, whereas the remaining 50.4% of them were resistant to one or more antimicrobial agents. Among them 7.0% were resistant to a single antimicrobial agent (streptomycin or tetracycline), and 20.0% were resistant to two antibiotics (ciprofloxacin and nalidixic acid or tetracycline and streptomycin). Multidrug resistance, defined as resistance to antimicrobial agents belonging to at least two different classes of antibiotics, was confirmed in 26.1% of the tested *Campylobacter* isolates There was found resistance to: - quinolone and fluoroquinolone antibiotics (nalidixic acid and ciprofloxacin) 38.3% (*C. jejuni* 29.4% and *C. coli* 51.1%); - streptomycin 24.3% (8.8% *C. jejuni* and 46.8% *C. coli*); - tetracycline 20.9% (16.2% *C. jejuni* and 27.7% *C. coli*); - erythromycin 4.3% (0.9% *C. jejuni* and 4.3% *C. coli*); - gentamicin 2.6% (0.9% *C. jejuni* and 1.7% *C. coli*) All of the Campylobacter isolates were sensitive to chloramphenicol. Multidrug-resistant strains constituted 13.2% and 44.7% of *C. jejuni* and *C. coli* isolates, respectively	Wieczorek et al. 2013 (PL)
*Salmonella*	Retail meat products samples: poultry meat, pork, beef, mixed meat, eggs	Geno-serotyping	disc-diffusion method	In total of 106 strains of *Salmonella* were collected from retail meat products in the framework of a control and monitoring programme carried out by sanitary-epidemiological stations across Poland. Strains isolated from poultry meat (81), pork (7), beef (3) and mixed meat (15) were serotyped and tested for resistance to 19 antimicrobial agents. The serological analysis identified 21 serotypes, including: *- Salmonella* Enteritidis (34.9%), *- Salmonella* Infantis (14.2%), *- Salmonella* Typhimurium (10.4%) Most *Salmonella* strains (73; 68.9%) were resistant to one or more antimicrobial agents. In this group: *-* 31 isolates were resistant to one antibiotic, *-* 4 isolates to 2 antibiotics, *-* 10 isolates to 3 antibiotics, *-* 15 isolates to 5 or more antibiotics Resistance to nalidixic acid (52.8%) was the most frequently observed, as was resistance to tetracycline (32.1%), ampicillin (28.3%), streptomycin (28.3%) and sulfonamides (26.4%). All of the tested strains were sensitive to cefepime, cefotaxime, ceftazidime, ceftriaxone, ciprofloxacin, ertapenem and imipenem. *Salmonella* strains isolated from poultry meat had the broadest spectrum of resistance (12 of 19 antimicrobials tested) compared to isolates from other sources. The results indicate that of the isolates tested: *-* 54% of *Salmonella* Enteritidis serotype were resistant to at least one antibiotic, but significantly higher levels of resistance have been reported for other serotypes: *- Salmonella* Newport 100%, *- Salmonella* Typhimurium 91%, *- Salmonella* Hadar 85.7%, *- Salmonella* Virchow 80%, *-* Salmonella Infantis 80% (Mąka et al., 2014) From retail eggs were isolated 25 strains of *Salmonella*, with as many as 84% of the *Salmonella* Enteritidis serotype. Drug resistance analysis showed that 17 isolates from eggs (68%) were resistant to one or two antibiotics	Mąka et al. 2015 (PL)
*Salmonella*	Samples of bulk tank milk from farms (n = 300)	serotyping by use HM serum for flagellar antigen and next confirmation of serotype by PCR	disc-diffusion method	The isolated strains were analysed for sensitivity to 7 antibiotics: erythromycin, gentamicin, ciprofloxacin, ampicillin, tetracycline, doxycycline and chloramphenicol. The study included 300 milk samples, of which 16 contained *Salmonella* bacteria, all belonging to the *S.* Enteritidis serotype. Drug resistance analysis showed that 100% of the isolates tested were sensitive to 3 out of 7 tested antibiotics: ciprofloxacin, gentamicin and chloramphenicol, while as many as 87.5% isolates were resistant to at least one of the remaining antibiotics tested: - 13 isolates were resistant to ampicillin, - 8 to erythromycin, - 1 to doxycycline, - 1 to tetracycline Furthermore, multidrug resistance was confirmed in 26% isolates. The high level of occurrence of antimicrobial resistance among *Salmonella* bacteria isolated from milk is best illustrated by the fact that only 2 out of 16 isolates tested (12.5%) were susceptible to all studied antibiotics	Wiszniewska-Łaszczych et al. 2018 (PL)
*Enterobacteriaceae*	Samples of fresh raw meat (poultry, pork, beef, mechanically minced meat) and processed meat (cured meats) intended for sale, obtained from meat processing plants (n = 433)	Microgen GN-ID system	ETESTs	In total of 114 *Enterobacteriaceae* strains were isolated, belonging to *Escherichia, Klebsiella, Serratia, Enterobacter, Proteus, Hafnia, Citrobacter, Salmonella* and *Shigella* genera. The bacteria were isolated mostly from raw meat, mainly minced beef. Similarly, in case of pork, the bacteria were isolated mostly from minced meat and in case of cured meats – from white sausages. Antibiotic susceptibility was tested for the following 10 substances: piperacillin, piperacillin with tazobactam, cefotaxime, cefuroxime, imipenem, ceftazidime, gentamicin, tobramycin, ciprofloxacin and trimethoprim with sulfamethoxazole. A total of 43 (37.7%) of the isolated strains were resistant to the tested antibiotics: - 10 *Echerichia coli* strains, - 9 *Serratia* strains, - 6 *Proteus* strains, - 4 *Salmonella* strains, - 4 *Klebsiella* strains, - 4 *Enterobacter* strains, - 3 *Shigella* strains, - 3 *Hafnia alvei* strains Most of these strains (28; 65%) showed resistance to one antibiotic, but as many as 15 isolates (35%) were resistant to at least 2 substances (9 strains to 2 antibiotics, 3 strains to 3 antibiotics and 1 strain each to 4 or more antibiotics). Resistance to cephalosporins, penicillins and trimethoprim with sulfamethoxazole was the most common	Szewczyk et al. 2019 (PL)
*Escherichia coli*	Strains of E. coli isolated from raw dairy milk (n = 19) and poultry waste (n = 20)	biochemical tests	disc-diffusion method	The isolated strains were tested on resistance to penicillins (ampicillin), aminoglycosides (gentamicin), phenicols (chloramphenicol), tetracyclines (tetracycline) and macrolides (mixture of sulfamethoxazole and trimethoprim). *E. coli* isolates from dairy raw material samples were characterised by a higher resistance to ampicillin and a higher susceptibility to the remaining antibiotics under study than isolates obtained from poultry waste. Isolates from dairy raw material samples showed the highest resistance to ampicillin (78% of isolates). It should be noted that the tested isolates were highly sensitive to the other antibiotics. 80 to 90% of isolates were susceptible to tetracycline, chloramphenicol and a mixture of sulfamethoxazole and trimethoprim, while 70% were found resistant to gentamicin*. E. coli* isolates from poultry waste showed the highest resistance to ampicillin and tetracycline (40–45.0%) and the lowest resistance to chloramphenicol (90.0%) and gentamicin (60%)	Krzyśko-Łupicka et al. 2017 (PL)
Meticiline-resistant *Staphylococcus aureus* (MRSA)	Nasal swabs were taken in 2 slaughterhouses with their own meat processing plants (n1 = 804 and n2 = 270) from pigs raised in 11 different farms (n = 1074) Pork meat samples were taken from two company shops (nm1 = 396 and nm2 = 140). The meat samples were collected within 4 days after slaughter at the latest	molecular	disk-diffusion method	In total of 420 *S. aureus* isolates were obtained, including: - 203 isolates from a total of 1074 swine nasal swabs, - 217 isolates from a total of 536 meat samples Of the total 420 *S. aureus* isolates, as many as 36 (8.6%) were classified as MRSA, all came from swine nasal swabs. All MRSA isolates were resistant to cefoxitin, oxacillin, penicillin and tetracycline and sensitive to gentamicin, erythromycin, ciprofloxacin, norfloxacin and vancomycin. All MRSA isolates came from pigs from one slaughterhouse and from a single farm. None had the *mec*C gene. All of them were classified as *spa* type t011 SCCmecV Analysis showed that 28 isolates (14%) of *S. aureus* from nasal swabs and 21 isolates (10%) from raw meat identified as borderline oxacillin-resistant (BORSA strains). All of them were sensitive to cefoxitin and amoxicillin with clavulanic acid. They all contained the *blaZ* gene and were *mecA* and *mecC* negative. Furthermore, *Spa* type t034 was found to prevail (64%) among BORSA strains from pigs and t091 was the most frequent among BORSA strains from meat (38%)	Krupa et al. 2015 (PL)
*Staphylococcus aureus*	Milk, butter and smoked meat samples from animal food processing plants	n/a	disk-diffusion method	The analysis showed of 7 *S. aureus* strains present in studied material. Only one of the strains was susceptible to all of the 18 antibiotics tested; 6 strains showed multidrug resistance, including 1 strain resistant to as many as 16 antimicrobial compounds. Among them 3 strains resistant to cefoxitin were identified as MRSA	Garkavenko et al. 2019 (UA)
*Salmonella*	Test samples were taken from heart, liver and stomach muscles of the broilers that died as a result of disease on an organic farm	biochemical tests	disk-diffusion method	From the pathological material were isolated bacteria *Salmonella* group D. The antibiogram results showed that the isolated bacteria were characterised by a significant resistance to a number of traditional antibacterial drugs used in human therapy. The isolates were resistant to drugs from the macrolide and lincosamide groups, methicillin and oxacillin from the penicillin group, cephalothin from the cephalosporin group, nalidixic acid and nitroxoline from the quinolone group, neomycin from the aminoglycoside group and vancomycin. At the same time, the isolated strains showed moderate resistance to chloramphenicol	Kucheruk et al. 2018 (UA)
*Salmonella enterica*	Pork meat production facility	molecular	molecular	The analysis of *Salmonella enterica* subsp. Enterica serovar Kottbus strain Kharkiv (serogroup C2-C3) revealed chromosomal fragments encoding antibiotic resistance	Arefiev et al. 2020 (UA)

*PL* Poland; *UA* Ukraine; *n/a* data not available

Wieczorek and Osek (2018) presented the results of a report published by European Food Safety Authority (EFSA) and European Centre for Disease Prevention and Control (ECDC) concerning, among others antibiotic resistance in *Campylobacter* isolates collected from animals, food and people in the EU in 2016. The study is based on Directive 2003/99/EC and Commission Implementing Decision 2003/652/EU on the basis of data submitted by EU Member States. The *Campylobacter* isolates discussed in the report demonstrated very diverse resistance depending on the species of bacteria and source of their isolation. A vast majority of serologically-determined (identified) isolates from people were *C. jejuni* and *C. coli*. Isolates obtained from people were tested for resistance to 5 antimicrobial substances (ciprofloxacin, amoxicillin/clavulanic acid, erythromycin, gentamicin and tetracycline). According to the authors, the average incidence of campylobacteriosis is 66.3/100,000 for EU residents and 2.0/100,000 for Poland residents. In comparison with other EU countries, the number is quite low. In 2016, 773 cases of campylobacteriosis were confirmed in Poland. Even so, the report lacks data about the antibiotic resistance of strains isolated from those people, because Poland did not provide such information. For the remaining EU Member States, which provided data (17 Member States), the largest number of resistant strains of *C. jejuni* were detected for ciprofloxacin (54.6%) and tetracycline (42.8%). *C. coli* strains were resistant mostly to tetracycline (64.8%) and ciprofloxacin (63.8%). It follows from the report that microorganisms belonging to *Campylobacter* are mostly found in poultry, and much less frequently in cattle or swine. Data on resistance to antimicrobial agents of *Campylobacter* strains from animals included *C. jejuni* and *C. coli* isolates. In order to assess antimicrobial resistance, 6 antibiotics were used (ciprofloxacin, erythromycin, gentamicin, nalidixic acid, streptomycin and tetracycline). For *C. jejuni* isolates obtained from broiler chickens, 164 out of 176 (93.2%) proved resistant to ciprofloxacin, whereas 150 strains were resistant to nalidixic acid. No erythromycin-resistant *C. jejuni* isolate was found in Poland and in 15 other countries. In Poland the percentages isolated from broilers strains of *C. jejuni* resistant to 4 of the 6 antibiotics taken into account (ciprofloxacin, streptomycin, tetracycline and nalidixic acid) were much higher than the average rates calculated for all EU countries. The number of *C. jejuni* strains isolated from turkeys in Poland was 174. Antimicrobial resistance analysis showed that 162 (93.1%) of strains were resistant to ciprofloxacin, 135 (77.6%) to nalidixic acid, 117 (67.2%) to tetracycline, 29 (16.7%) to streptomycin and 1 (0.6%) to erythromycin. The percentages of strains resistant to nalidixic acid, tetracycline and streptomycin were higher than the average rates for EU countries. The rest of the data did not differ considerably from the information reported from other countries or were not reported at all (Wieczorek and Osek 2018).

The prevalence of antibiotic-resistant strains of microorganisms in food suggests that their role in the transmission of genes coding for resistance to antimicrobial substances may be much more important than previously thought. A significant problem is the resistance of *Enterococcus* bacteria isolated from food to antibiotics used to treat humans. Reports indicate that enterococci isolated from ready-to-eat foods of animal origin present a resistance profile very similar to strains isolated from raw pork, beef or poultry meat. Food may be a source of antibiotic-resistant gram-positive cocci carrying genes that determine horizontally spreading resistance. Antibiotics, whose use as growth promoters is now banned in Europe, are still widely used in veterinary medicine for preventive and therapeutic purposes (Chajęcka-Wierzchowska et al. 2017).

In Ukraine Garkavenko et al. (2019) studied the prevalence of drug resistance in seven strains of *Staphylococcus aureus* bacteria isolated from samples obtained from meat processing plants from different regions of Ukraine. The analysis showed that only one of the strains was susceptible to all of the 18 antibiotics tested. It was found that 6 strains showed multidrug resistance, including 1 strain resistant to as many as 16 antimicrobial compounds. A total of 3 strains resistant to cefoxitin were identified as MRSA (Garkavenko et al. 2019).

Kucheruk et al. (2018) studied fallen broilers that had died from a disease. The broilers came from an organic farm. The aim of the study was to determine the cause of death and identify the causative agent of the chicken disease, as well as to determine the antibiotic susceptibility of the strain of the microorganism isolated from the pathological material. Test samples were taken from heart, liver and stomach muscles of the dead animals. Drug resistance was determined by the disk-diffusion method. Group D *Salmonella* bacteria were isolated from the pathological material. The antibiogram results showed that the isolated bacteria were characterised by a significant resistance to a number of traditional antibacterial drugs used in human therapy. The isolates were resistant to drugs from the macrolide and lincosamide groups, methicillin and oxacillin from the penicillin group, cephalothin from the cephalosporin group, nalidixic acid and nitroxoline from the quinolone group, neomycin from the aminoglycoside group and vancomycin. At the same time, the isolated strains showed moderate resistance to chloramphenicol (Kucheruk et al. 2018).

Arefiev et al. (2020) sequenced the complete genome of *Salmonella enterica* subsp. Enterica serovar Kottbus strain Kharkiv (serogroup C2-C3), which was isolated from a commercial pork production facility in Kharkiv, Ukraine. The analysis revealed chromosomal fragments encoding antibiotic resistance. The authors drew attention to the risk of contamination of this bacteria farm animals and humans (Arefiev et al. 2020).

### The results of epidemiological research concerning the prevalence of antibiotic-resistant zoonotic bacteria in the vicinity of breeding farms

Wychodnik et al. (2020) conducted a study aimed at verifying whether the intensive breeding of poultry (broilers) affects the level of contamination of the soil that surrounds poultry houses, and if so to what extent. The study evaluated the prevalence of antibiotic-resistant bacteria strains, as well as the presence of 26 selected pharmaceutical agents in the soil surrounding the farms. The study was conducted twice, in March and July, on a farm located in one of the municipalities in the north-east of Poland. Samples were collected from a poultry house that had been cleaned and disinfected after each production cycle. During breeding, the necessary amount of antibiotics (such as amoxicillin, doxycycline, enrofloxacin) is being administered ad hoc through water troughs. According to the authors, the water line is not cleaned after the water with the drug is poured through it, but the waiting period for antibiotics is observed. On the farm, soil samples were taken within the distance of 1, 10 and 50 m from the poultry house, one sample was taken directly outside of the house (a place where used litter is stored) and two reference samples were collected within a 450 and a 1000-m distance (outside of the farm). Samples were tested for antimicrobial agents using highly efficient liquid chromatography. Using the disc-diffusion method, the bacteria isolated from soil samples were tested for resistance to 5 antibiotics (trimethoprim, sulfamethazine, tetracycline, spectinomycin, ciprofloxacin). The analysis revealed that 7 and 4 of the 26 tested substances were present in the soil samples collected from the farm in March and April, respectively. The sample taken in March from the place where used litter is stored was the most contaminated one (6 substances). In the reference samples**,** only 2 substances were detected, sulfanilamide and paracetamol (which are not antibiotics). Said substances were not found in any other sample. Moreover, concentration levels of sulfamethazine and sulfanilamide in samples collected in July were about 5 times higher than in those taken in March. Regarding bacteria isolated from the soil samples, the analysis covered 66 isolates collected from the farm, 15 isolates from the sample taken 450 m away from the farm and 4 isolates from the sample taken 1000 m away from the farm. Across the strains, the highest resistance was observed for sulfamethazine and trimethoprim, with only 6 and 7 isolates from the soil collected from the poultry farms, respectively, susceptible to those drugs—the remaining isolates were found resistant. In the soil sample taken 450 m away from the farm, the number was even lower since there were only two strains that were susceptible to trimethoprim, and no strains susceptible to sulfamethazine. Within a 1000-m radius from the farm, there were no strains susceptible to the above-mentioned substances. All strains from each type of soil were susceptible to ciprofloxacin. Considering the results obtained for 85 bacteria isolates coming from different areas surrounding the poultry house, no significant difference in the level of resistance evaluated for various distances from the farm was found (Wychodnik et al. 2020).

In recent years, more and more attention has been drawn to a bacteria belonging to the *Staphylococcus aureus* species, referred to as LA-SA (livestock-associated *S. aureus*), due to its ability to colonise farm animals and transfer to people. Mroczkowska et al. (2017) conducted a study to determine the spread of LA-SA on selected pig farms across Poland, characterise the population structure of the *S. aureus* and estimate the prevalence of LA-SA across farmers and veterinarians that have contact with animals. Samples were collected on 123 pig farms located in 15 out of 16 Polish voivodeships, between August 2010 and November 2012. The samples included 321 nasal swabs (of which 283 were taken from farm owners and workers and 38 from veterinarians working on the farms), 1845 nasal swabs from pigs, and 1845 dust samples (in the form of swabs, mainly from pens and ventilation ducts). All analyses were conducted using molecular methods. The isolated *S. aureus* strains were analysed for methicillin resistance in accordance with EUCAST guidelines. *S. aureus* bacteria were isolated on 79 farms (64.2%) in 14 voivodeships, while the presence of LA-SA was confirmed on 71 of them. Moreover, LA-SA was identified on 18 farms, in samples collected from the swabs taken from workers. Among the *S. aureus* isolates found in people, 50 were from pig breeders and 11 from veterinarians. LA-MRSA were present in samples taken from 26 farms, and LA-MSSA were found in samples taken from 53 farms. LA-MRSA were found in 5 isolates derived from farmers and 7 isolates derived from dust.

A total of 190 *S. aureus* isolates were identified: 72 (38%) MRSA and 118 (62%) MSSA. Isolates were divided into 10 genetic lines:

- CC398 (72.6% of all isolates)
- CC9 (13.2%)
- CC30 (7.9%)
- CC22 (1.6%)
- CC1, CC8, CC15 (1.1% each)
- CC5, CC12 and CC182 (0.5% each).

Four of these lines, CC5, CC8, CC15, and CC182, were found only among isolates collected from people. Most of the *S. aureus* isolates (36; 72.0%) identified in pig farmers belonged to the CC398 genetic line, the remaining ones represented CC1, CC5, CC8, CC9, CC15, CC22, CC30, and CC182. Isolates obtained from veterinarians were classified into three genetic lines: CC398, CC9, and CC15. LA-SA isolates collected from farmers were marked at 13.2% (38/283), CC398 carrier at 12.7% (36/283), and ST398-MRSA carrier at 3.2% (9/283) while for veterinarians these values were: 21.1% (8/38), 18.4% (7/38), and 10.5% (4/38), respectively. All MRSA isolates (*n* = 13) identified among the human population belonged to ST398. It is stated by the authors that ST398 carrier among Polish farmers is low in comparison with results obtained from the USA and Canada (about 20%), Belgium, Germany, and Denmark (over 80%). Carrier among Polish veterinarians is at a level similar to Belgians and lower than Germans (45%) (Mroczkowska et al. 2017). With the exception of Asia, the most common LA-MRSA strain in the world is the one with sequence type (ST) 398 (Schnitt et al. 2020).

Popowska et al. (2012) analysed different soil types for antibiotic-resistant bacteria and resistance genes in soil.

Tests were conducted on five types of soils and compost.

- compost derived from composting plant residue (manure and/or antibiotics were not applied),
- soil from a pine forest (manure and/or antibiotics were not applied),
- soil from a vegetable garden (manure was used),
- soil from an apple orchard,
- soil from fruit orchards combination,
- soil from arable land.

Soil for intensive fruit and vegetable farming were collected from cropping systems, where antibiotics (streptomycin and oxytetracycline) have been applied. Manure originated from animals that had been administered erythromycin. The analysis of antibiotic resistance of bacteria was conducted using the MIC method, according to EUCAST guidelines. For gene resistance marking, the PCR method was applied. The most-frequently found resistance genes were:

- *tet*(B), *tet*(D), *tet*(O), *tet*(T), *tet*(W) for tetracycline resistance,
- *str*(A), *str*(B), *aac* for streptomycin resistance,
- *erm*(C), *erm*(V), *erm*(X), *msr*(A), *oler*(B) i *vga* for erythromycin resistance.

In soil bacteria isolates, genes characteristic for transposons Tn916, Tn1549, TnB1230, Tn4451, and Tn5397 were identified. Composted and forestry soils were characterized by a lower level of antibiotic resistance than agricultural and garden soils. Multidrug-resistant bacteria were detected only in soil deriving from vegetable garden, which also had the highest immunity level to three classes of antibiotics (tetracycline, erythromycin, streptomycin). The soils untreated with manure had higher amounts of antibiotic-resistant bacteria but these were characterized by lower MIC values, had less antibiotic resistance genes and were not multidrug-resistant. Bacteria with highest MIC values were detected in soil treated with manure or in agricultural system soils, with a history of antibiotic application (Popowska et al. 2012).

Izdebski et al. (2016) described a case of a 50-year-old woman, who, in 2015, was submitted to a hospital in the north of Poland, with pneumonia, diarrhea and urinary tract infection. *Escherichia coli* bacteria, that is resistant to colistin (MCR-1- *eng. mobilized colistin resistance)* was isolated from the patient’s urine. The isolated strain’s resistance was marked using MIC method, and the presence of resistant genes using PCR method. The bacteria were demonstrating resistance to a number of other antibiotics (penicillin, amoxicillin/ clavulanic acid, cephalosporin—with the exception of cefepime, aztreonam, fluoroquinolones, tetracycline and co-trimoxazole). It is suspected that it was a zoonotic bacteria, because the patient, due to her place of living, could have had contact with farming animals. It was a first, described case of MCR-1 positive bacteria in Poland, whilst such cases have already been confirmed in the world (Europe, Asia, USA) (Izdebski et al. 2016).

Bukowski et al. (2019) determined complete plasmid sequences derived from 18 poultry *S.* *aureus* strains. They analyzed the frequency of occurrence of antibiotic and heavy metal resistant determinants and virulence gene factors**.** The obtained results indicate that the most of plasmids that occur in tested strains have already been described before and they were found in clinical isolates’ strains, typical for human or humane of animal origin**.** It has been proven that livestock-related *Staphylococcus* are a significant reservoir of factors determining antibiotic resistance, and moreover, that transmission of those genes from animals to humans is possible (Bukowski et al. 2019).

It worth notice that in Ukraine there was not available literature on the field of epidemiological studies concerning the presence of antibiotic-resistant zoonotic bacteria in the vicinity of breeding farms.

## Discussion

This review showed that livestock, animal manure and animal products are the source of many bacterial antibiotic resistance genes. The authors demonstrated the presence of the genes of resitance to various groups of antibiotics. Many of the isolated bacteria strains showed multi-drug resistance. The most frequently detected antibiotic resistance genes were: tet, str, erm, mec, bla, cat, qnr.

Currently subsequent legal actions, aimed at diminishing further spreading of antibiotic resistance across the environment, are being taken at both national and European Union level. The aim of this law is to change the growing, antibiotic resistance spreading trend in the environment of large-scale intensive animal farming by stricter regulation to mitigate the antibiotics usage in livestock. Although, it needs to be clearly stated that the only chance of slowing this process down is cooperation between authorities, the world of science and animal breeders. Beside the selective pressure, there is a mechanism of horizontal gene resistance transfer between, even unrelated, bacteria species. Thus the first step towards improving the situation, seems to be an overall increase of social awareness in regard to antibiotic usage, the problem concerns not only the abuse of these substances in veterinary medicine, but also in medicine. That is the reason why not every case of antibiotic-resistant bacteria strain infection across residents of even highly intensified animal farming areas, will be connected with emissions released from these objects. With such a small number of studies conducted in farm areas it is difficult to conclude even a roughly estimated level of health risk related to animal bacteria antibiotic resistance, that inhabitants are exposed to. Especially, since these are usually rural territories, there is a certain percentage of inhabitants that run their own farms and are in current contact with their animals. Currently, subsequent actions, aimed at diminishing further spreading of antibiotic resistance across the environment, are being taken at both national and European Union level. The aim of this law is to change the growing, antibiotic resistance spreading trend in the environment of large-scale intensive animal farming by stricter regulation and limitation of antibiotics usage in livestock. Although, it needs to be clearly stated that the only chance of slowing this process down is cooperation between authorities, the world of science and animal breeders. Beside the selective pressure, there is a mechanism of horizontal gene resistance transfer between, even unrelated, bacteria species. The first step towards improving the situation, seems to be an overall increase of social awareness in regard to antibiotic usage, the problem concerns not only the abuse of these substances in veterinary medicine, but also in medicine. That is the reason why not every case of antibiotic-resistant bacteria strain infection across residents of even highly intensified animal farming areas, will be connected with emissions released from these objects. With such a small number of studies conducted in farm areas it is difficult to conclude even a roughly estimated level of health risk related to animal bacteria antibiotic resistance, that inhabitants are exposed to. Especially, since these are usually rural territories, there is a certain percentage of inhabitants that run their own farms and are in current contact with their animals.

## Conclusions

### Limitations

Although matters related to the usage of antibiotics in animal farming have been regulated by law in EU and their execution is in Poland under strict supervision provided by an assigned inspection, the curve that represents bacterial mutation towards antibiotic resistance is still rising. It is difficult to compare the scale of multidrug-resistant bacteria (MRSA, ESBL) dissemination in Poland and Ukraine with other EU countries due to lack of more extensive studies and large-scale monitoring in these two countries. A series of works concerning resistance of pathogenic bacteria isolated from intensive animal farming environment have been published in Poland. However, these studies have been conducted on single farms and with a very limited number of trials, often selected in a deliberate manner and without a genotypic drug resistance marking, which in turn might be the cause of overestimating the proportion of positive results. The data accessible from Ukraine need to be considered almost negligible, but equally disturbing.

1. The literature review does not allow for unambiguous assessment of the health risk caused by antibiotic-resistant bacteria emitted to the environment by intensive livestock facilities not only in Poland and Ukraine, but more generally as well. Based on the literature review, it is possible to indicate some potential paths of bacteria spreading to the environment, as air (bioaerosols released from farms), soil and water, contaminated with bacteria derived from animals’ excrements and people (farms employees and staff), wild animals (rodents and birds) and raw foodstuffs of animal origin (with the exception of pasteurized food). Several reports concerning identification of antibiotic-resistant bacteria in foodstuffs of animal origin (e.g. raw meat or eggs) should be treated as a barometer of a spreading scale of the ARB in animal production, and less within the category of actual risk for consumers health. With good hygiene and proper thermal treatment these products cause limited risk of infection, although of course this problem should be monitored on a much larger scale than before. Certainly, intensive animal farming shows potential for pathogenic ARB emissions to the environment, however, an assessment of the effect it might have on actual morbidity of people living in farms vicinity the current stage of knowledge is difficult to estimate.
2. The risk of releasing antibiotic-resistant bacteria to the environment, does not only concern animal farming, but also other facilities of animal origin food supply chains, especially slaughterhouses.
3. Environmental exposure of people living in the vicinity of animal farms to bacteria emitted by large-scale intensive animal farms may cause risks of a local infection (e.g. wound or cut) or more systemic (e.g. respiratory, urinary or digestive system or even sepsis). Among people living in the livestock vicinity are also highly susceptible populations (mainly children, elderly and people with decreased levels of immunity). Thus there is a necessity to legal regulate of the distance from the farm facilities to the nearest residential buildings. The solution worth to consider is a focus on limit values (norms) for multidrug-resistant pathogen bacteria present in the air around farms as a base to assign the distance with low health risk. In order to correctly and reliably assess this distance are needed the results of extensive research conducted in national conditions. This way of assessing the safe distance would help reduce the health concerns among people living around livestock facilities.

## Data Availability

All data generated or analysed during this study are included in this article.
